# Prevalence of breast, cervical, and colorectal cancer screenings among select New York City populations

**DOI:** 10.1186/s12885-025-14763-z

**Published:** 2025-09-30

**Authors:** Laura C. Wyatt, Charlie H. Nguyễn, Madison N. LeCroy, Victoria Foster, Julie Kranick, Yousra Yusuf, Sonia Sifuentes, Chau Trinh-Shevrin, Simona C. Kwon

**Affiliations:** 1 Department of Population Health, NYU Grossman School of Medicine, New York, NY 10016 USA; 2 Bay Area Cancer Connections, San Mateo, CA USA

**Keywords:** Cancer screening, Mammography, Papanicolaou test, Colonoscopy, Ethnic and racial minorities, Urban population, Resource-Limited settings

## Abstract

**Background:**

Prior studies have found racial and ethnic disparities in cancer screenings, yet smaller minority ethnic groups are often aggregated.

**Methods:**

Data from the 2021–2022 Community Health Resources and Needs Assessment (Cancer CHRNA) and the 2017–2020 NYC Community Health Survey (CHS) examined the prevalence of breast, cervical, and colorectal cancer screenings among Eastern European, Afro-Caribbean, Latine, Chinese, Korean, South Asian, and Southwest Asian and North African (SWANA) groups in New York City. Multivariable logistic regression models estimated adjusted relative risks of cancer screening outcomes by group.

**Results:**

Up-to-date mammogram screening was low (< 70%) among all groups except Afro-Caribbean in the Cancer CHRNA; and among South Asian, Chinese, and Eastern European groups in the CHS. In logistic regression, South Asian and SWANA groups were less likely to have received an up-to-date mammogram compared to the Afro-Caribbean group in the Cancer CHRNA; no group differences were found in the CHS. Up-to-date Pap screening was low (< 70%) among all groups except Latina in the Cancer CHRNA; and among South Asian and Chinese groups in the CHS. In logistic regression, all other groups were less likely to have received an up-to-date Pap test compared to the Latina group in the Cancer CHRNA; and Chinese and South Asian groups were less likely to have received an up-to-date Pap test compared to the Latina group in the CHS. Up-to-date colonoscopy screening was low (< 70%) among all groups in the Cancer CHRNA; and among SWANA, South Asian, Chinese, and Eastern European groups in the CHS. In logistic regression, all groups except Chinese were less likely to have received an up-to-date colonoscopy compared to the Eastern European group in the Cancer CHRNA; and the Chinese and SWANA groups were less likely to have received an up-to-date colonoscopy compared to the Afro-Caribbean group in the CHS.

**Conclusions:**

Disparities in cancer screenings differed by screening type and survey, with larger disparities found among groups in the Cancer CHRNA. System level efforts are needed to monitor cancer screening disparities by disaggregating diverse groups; culturally tailored strategies should be used to raise awareness to increase screening.

**Clinical trial information:**

Not applicable.

**Supplementary Information:**

The online version contains supplementary material available at 10.1186/s12885-025-14763-z.

## Background

Screening is an important early detection tool for cancers. Cancer screenings are generally lower among non-White racial and ethnic groups compared to White groups [1–4], and disaggregated data can identify disparities to better understand the needs of underserved groups and set priorities to increase cancer screening [5].

In New York City (NYC), colonoscopy screening rates have increased since 2003 for White, Black, and Latine groups—largely due to an initiative to increase timely colonoscopy screening [6, 7]. However, screening rates for Asian American groups in NYC have lagged behind White and Black subgroups [7–9]. In 2017, the NYC Community Health Survey (CHS) showed the rate of colonoscopy screening for adults aged 50 and older to be 72% for Black, 69% for Latine, 69% for White, and 65% for Asian and Pacific Islander (PI) groups [7]. Colonoscopy screening rates also differed by neighborhood; residents of Manhattan had a higher colonoscopy screening rates compared to diverse, lower income Brooklyn neighborhoods [7]. Data from the 2022 NY Behavioral Risk Factor Surveillance Survey (BRFSS) found the lowest prevalence of CRC screening (receiving at least one of the recommended tests among individuals ages 45–75) for the non-Hispanic (NH) American Indian/Alaska Native (40.9%) and NH Asian American and PI (49.8%) individuals, followed by Hispanic (64.7%), NH White (72.6%), NH Black (73.4%) individuals [10].

While the NYC Community Health Survey (CHS) collects colonoscopy screening rates every year, Pap and mammogram screening data is collected more sporadically. Pap screening was last collected in 2017, and mammogram screening was last collected in 2019. A 2011 report from the NYC Department of Health and Mental Hygiene (DOHMH) found that among women aged 40 and over, Black (81%) and Latina (84%) women were more likely than White (75%) and Asian (76%) women to have received a mammogram in the past two years. Similar results were shown for cervical cancer screening: Black (81%) and Latina (84%) women aged 18 and over were more likely to have been screened in the past three years compared to White (77%) and Asian (68%) women [11]. Data from the 2020 NY BRFSS found the lowest prevalence of a Pap test in the past three years (no hysterectomy) among women ages 21–65 for NH Asian and PI women (63.6%), followed by Hispanic (79.9%), NH White (82.4%), and NH Black (83.2%) women [12]. Data from the 2022 NY BRFSS found the lowest prevalence of a mammogram in the past two years among women ages 40 and over for NH Asian and PI (69.3%) women, followed by NH White (73.1%), Hispanic (81.4%), and NH Black (83.7%) women [13].

While large health surveys often include broad racial and ethnic groups of White, Black, Latine, and Asian individuals, analyses are not always disaggregated by subgroups or often report only White, Black, Hispanic, and Other. When included, the Asian American group is often aggregated with the Native Hawaiian and Pacific Islander (NH/PI) group, masking potentially differences. Additionally, smaller groups such American Indian and Alaska Native (AIAN) are often aggregated into an “other” group, combining racial and ethnic categories with small sample sizes [14, 15]. Other detailed groups often disappear into larger racial and ethnic groupings, such as Southwest Asian and North African (SWANA) individuals, who are often grouped within the White racial or ethnic group, despite members of this group often identifying as another race or ethnicity, as well as Asian American groups (e.g., Chinese, Korean, South Asian), Hispanic groups (e.g., Mexican, Puerto Rican, Dominican), Black groups (e.g., African American, Caribbean, Sub-Saharan African) and White groups (e.g., Eastern European) [16].

In the large metropolitan city of NYC, immigrants account for a substantial proportion of the population (37.5%) [17]. Given the limited publications of recent data on cancer screenings in NYC using more detailed racial and ethnic breakdowns, more recent data is needed to identify and explain potential screening disparities among diverse NYC racial and ethnic groups. The current study investigates up-to-date colonoscopy, cervical cancer, and breast cancer screenings among seven distinct ethnic groups in NYC (Eastern European, Afro-Caribbean, Latine, Chinese, Korean, South Asian, and SWANA). We use data from the 2021–2022 Cancer Community Health Resources and Needs Assessment (Cancer CHRNA) as well as 2018 (mammogram), 2017 (Pap test), and 2018–2020 CHS data, two surveys that sample diverse ethnic groups in NYC. Up-to-date screenings are adjusted by ethnic group, socio-demographics, health care access, and language/nativity. By analyzing and comparing the Cancer CHRNA and CHS datasets, which have different sampling modalities, we are able to see if the detailed ethnic groups that are included in the analyses show similar or different results, as well as allowing for validation of the community screening method used in the Cancer CHRNA.

## Materials & Methods

### Cancer CHRNA

The Perlmutter Cancer Center (PCC) at NYU Langone Health (NYULH) collaborated with 23 community partners to develop the Cancer CHRNA, a cross-sectional survey to document self-reported socio-demographics and cancer screening knowledge, attitudes, behaviors and resources available among racial and ethnic minoritized and immigrant populations in the PCC catchment area (lower Manhattan, Brooklyn, Queens, and parts of Nassau and Suffolk counties). Questions were taken from other sources, including the BRFSS [18], the Health Information National Trends Survey (HINTS) [19], and the PhenX Toolkit [20] (see additional file 1). The survey was administered between October 2021-December 2022, with a final dataset of 2,636 respondents.

To reach minoritized immigrant populations, participants were recruited through in-person clinical and highly frequented community-based settings such as nail salons, halal trucks, and at community events, as well as through virtual recruitment via partner-generated client lists, and flyers placed at community locations. Each study community health worker (CHW) was assigned one to two clinical partners where they were embedded for our clinically based recruitment. Similarly, each CHW was paired with one to two community-based organizations that serve the community they represented. Furthermore, CHW managers worked with CHW to design a culturally tailored plan for reaching people in additional locations to ensure recruitment was inclusive of populations that are not often included in health research; these locations varied by target population. For instance, halal trucks were included for the SWANA population, while Spanish language senior centers were a focus for Latine recruitment. Recruitment materials included a website URL, QR code, and contact information for multilingual staff.

Eligibility criteria included aged ≥ 18 years of age, catchment area residence, and speaking a survey administration language. A translation company translated the survey from English into Arabic, Bangla, Simplified and Traditional Chinese, Haitian Creole, Korean, Russian, Spanish, and Urdu. CHWs checked and edited translations as needed. Approximately 38% of individuals were surveyed in-person by CHWs, who simultaneously entered the data into REDCap, while 62% were surveyed directly online through REDCap [21, 22]. The study was approved by the NYULH Institutional Review Board (IRB).

### New York City Community Health Survey (CHS)

The CHS is a population-based telephone survey conducted annually by the DOHMH to provide data on the health of New Yorkers. Each year, approximately 10,000 NYC adults aged ≥ 18 years are surveyed on approximately 125 questions, covering general health status and mental health; health care access; nutrition and physical activity; smoking; cancer screening; and other health topics. While questions change across years (including cancer screening-related questions), a core group of demographic variables are included every year. The survey is conducted in English, Spanish, Russian, Chinese, Bengali, and Haitian Creole by telephone; Bengali and Haitian Creole were added in 2018. A computer-assisted telephone interviewing system is used to collect the survey data from individuals living in a household with a landline or who have a cellular telephone, using a list of telephone numbers provided by the commercial vendor. Among agreement to participate in the survey, one adults is randomly selected from the household to complete the survey [23]. Survey data for the combined years 2017–2020 was obtained via a data use agreement and was considered exempt from IRB review per self-certification.

### Measures

#### Outcome: cancer screening

The outcome of up-to-date breast cancer screening included self-report of a mammogram < 2 years ago among women aged 40–75 years, following recent US Preventive Services Task force (USPSTF) and the American College of Obstetricians and Gynecologists (ACOG) guidelines, which have recently changed to include initiation at age 40 rather than age 50 [24–26]. However, neither survey assessed mastectomy.

The outcome of up-to-date cervical cancer screening included self-report of a Pap test < 3 years ago among women aged 21–65 years based on USPSTF and ACOG guidelines [27, 28]. However, neither survey assessed human papilloma virus (HPV) testing nor hysterectomy.

The outcome of up-to-date colonoscopy screening included self-report of a colonoscopy < 10 years ago among adults aged 50–75 years based on previous USPSTF guidelines; this age recommendation, rather than the new guidelines for age 45–75 years, was used due to the years of CHS data collection (prior to 2022) [29, 30]. Additionally, CHS data only included up-to-date colonoscopy (< 10 years ago) among adults, and no other screening modalities for CRC; for consistency across datasets, colonoscopy screening was examined as the CRC screening outcome in the present analysis. A separate analysis was run for Cancer CHRNA data, which included an outcome of any up-to-date CRC screening test within the recommended time frame (colonoscopy < 10 years ago, sigmoidoscopy < 5 years ago, or fecal occult blood test [FOBT] test in the past year) according to USPTF guidelines. Stool based tests such as FOBT are convenient for average risk individuals because they can be done at home, however they must be done more frequently than the other tests [31].

For each cancer screening outcome, variables were dichotomized into yes (screened within the recommended time) and no/don’t know (not screened within the recommended time, never been screened, or don’t know).

#### Race and ethnicity

The Cancer CHRNA asked “What is your race or ethnic background?” with the following response categories (check all that apply): White; Hispanic, Latina/o, or Spanish origin; Black; Middle Eastern or North African; NH/PI; Asian; AI/AN, First Nations, Indigenous People of the Americas, or Alaska Native; and Some other race or origin (please specify). Each selection included an additional branching question for detailed ethnic group (e.g., Chinese, Korean, Bangladeshi, etc. for Asian subgroup; see Additional file 2). Country of birth, self-reported language spoken at home, and survey completion language were used to impute race and ethnicity when these questions were skipped. To place participants into appropriate groupings, some write-in responses were recategorized (e.g., White other: ‘Syrian’ was moved to SWANA: Syrian).

The CHS-calculated race and ethnicity variable included combined categories from two questions: (1) “Are you Hispanic or Latino?” and (2) “Which one or more of the following would you use to describe yourself as…” Response categories included White, NH; Hispanic; Black, NH; Asian/PI, NH; North African/Middle Eastern, NH (added in 2019); and Other, NH. A separate question assessed detailed Black race/ethnicity beginning in 2019, and additional questions assessed detailed Asian and Hispanic ethnicities (asked all years). Central/Western Asian and Eastern European ethnicities were determined using country of birth only; Latine race/ethnicity was determined using detailed race/ethnicity; Afro-Caribbean race/ethnicity included individuals reporting “Caribbean or West Indian” ancestry and Black individuals born in Caribbean countries; Chinese and Korean ethnicities included Asian American individuals reporting the respective Asian American ancestry as well as Asian American individuals born in China or Korea, respectively, who did not report detailed race/ethnicity; South Asian race/ethnicity included individuals reporting a South Asian race/ethnicity and a South Asian country of birth; and Indo-Caribbean race/ethnicity included individuals reporting a South Asian race/ethnicity and a Caribbean country of birth (see Additional file 2 for detailed questions).

Final groups for the Cancer CHRNA include Eastern European, Afro-Caribbean, Latine, Chinese, Korean, South Asian, and SWANA. Final groups for the CHS include Eastern European, Afro-Caribbean, Latine, Chinese, South Asian, and SWANA; SWANA is included for colonoscopy screening only due to sample size, and Korean was excluded from all outcomes due to sample size. The term SWANA is used rather than MENA, because it was believed to be a more accurate geographical representation of this group; MENA and SWANA are often used interchangeably [32].

### Socio-demographics

Age categorizations were used, because the CHS dataset did not include a continuous age variable. Age groups for breast cancer screening include: 40–44, 45–64, and 65–75 years; age groups for cervical cancer screening include 21–24, 25–44, and 45–65 years; and age groups for CRC screening include: 50–64 and 65–75 years. CHS age cut points end at 64 and 74, due to the shared categorizations. Additional socio-demographic variables included: educational attainment (< high school, high school, some college/technical school, and college graduate), annual household income (<$20,000, $20,000-$74,999, ≥$75,000, and don’t know/decline to state) for Cancer CHRNA, and poverty group (< 200%, 200–399%, and≥400%) for CHS.

### Access to care

Access to care included insurance status (private/other type, public, and none), a doctor’s visit in the past year for Cancer CHRNA, and having a regular health provider for CHS.

### Acculturation proxies

Nativity was assessed by being born in the US; US territories were considered US-born for the CHS and foreign-born for the Cancer CHRNA. Language was measured using the survey administration language (English or a non-English language) for Cancer CHRNA only; survey language variables were not included on the CHS dataset.

### Statistical analysis

We describe participant characteristics by ethnic group by dataset for: (1) women aged 40–75 years answering the mammogram screening questions; (2) women aged 21–65 years answering the Pap screening questions; and (3) individuals aged 50–75 years answering the colonoscopy screening questions. For Cancer CHRNA, unweighted frequencies were run for participant characteristics and cancer screening outcomes; ns and percentages are presented for participant characteristics and cancer screening outcomes, while stratified by ethnic group.

For CHS data, weights were applied to each record, consisting of an adjustment for the probability of selection and post-stratification weight. Post-stratification weights were created by weighting each record to the population of 34 United Hospital Fund (UHF) neighborhoods, accounting for respondents’ age, gender, and race/ethnicity [23]. Weighted estimates and 95% confidence intervals (CIs) were run for participant characteristics and cancer screening outcomes, while stratifying by ethnic group, following CHS data reliability guidelines [33].

Multivariable relative risk regression was performed to determine if racial and ethnic groups were associated with timely Cancer screenings, after adjusting for socio-demographic, health, and acculturation proxy variables. Reference groups were chosen for each outcome based on the group with the highest cancer screening rates within each dataset and cancer screening outcome; The adjusted relative risk (RR) of each up-to-date screening (mammogram, Pap test, and colonoscopy) was calculated across categories of predictors by estimating two regression models. Model 1 included ethnic group and age group (gender was included for colonoscopy screening). Model 2 included the variables from Model 1, as well as education, income or poverty group, health insurance, recent doctor’s visit or regular health care provider, and nativity. English language proficiency was included for Cancer CHRNA only. RRs and 95% confidence intervals are presented. For Cancer CHRNA data, SAS was used for analysis using proc genmod and Poisson distribution. For CHS data, SAS-callable SUDAAN was used to account for the complex sampling design.

## Results

### Breast cancer screening – Cancer CHRNA

In the Cancer CHRNA, *n* = 737 women respondents were eligible for breast cancer screening, answered mammogram screening questions, and identified as one of the included ethnic groups (Table 1). Up-to-date mammogram screening was lowest among SWANA individuals (50.6%), followed by South Asian (54.8%), Korean (63.4%), Latina (69.0%), Chinese (69.6%), Eastern European (72.5%), and Afro-Caribbean (73.2%) individuals. A larger proportion of individuals age 65–75 was shown for Chinese (42.8%) and Korean (31.2%) women. Over half of Chinese women (54.5%) had < high school education, while over half of Korean women (58.0%) and 46.2% of Eastern European women were college graduates. Household income was lowest (<$20,000) among SWANA (54.7%) and Chinese (38.7%) women. Latina (23.2%), Korean (23.3%), Afro-Caribbean (16.4%), and South Asian (13.6%) women were largely insured. Korean (28.9%), Latina (25.9%), SWANA (25.0%), and Afro-Caribbean (23.2%) were least likely to have visited a doctor in the past year. Greater than 93% of all groups were born outside of the US, except for Latina women (76.1%). Speaking English not well or not at all was highest among Chinese (75.2%), Korean (73.6%), and Latina (53.9%) women.

**Table 1:** Characteristics by racial/ethnic group, females ages 40–75 (Cancer CHRNA 2021–2022), n (%)

	Eastern European (*n* = 79)	Afro-Caribbean (*n* = 56)	Latina (*n* = 138)	Chinese (*n* = 225)	Korean (*n* = 89)	South Asian (*n* = 61)	SWANA (*n* = 89)
Mammogram < 2 years ago	58 (72.5)	41 (73.2)	96 (69.0)	158 (69.6)	59 (63.4)	34 (54.8)	45 (50.6)
Age, categories							
40–44	10 (12.5)	10 (17.8)	32 (23.0)	39 (17.2)	9 (9.7)	13 (21.0)	18 (20.2)
45–64	47 (58.7)	36 (64.3)	93 (66.9)	91 (40.1)	55 (59.1)	44 (71.0)	49 (55.1)
65–75	23 (28.8)	10 (17.9)	14 (10.1)	97 (42.8)	29 (31.2)	5 (8.0)	22 (24.7)
Education							
< High school	3 (3.8)	11 (20.0)	48 (34.8)	122 (54.5)	3 (3.4)	18 (29.0)	24 (27.0)
High school	14 (17.5)	10 (18.2)	24 (17.4)	42 (18.7)	20 (22.7)	11 (17.8)	9 (10.1)
Some college	26 (32.5)	15 (27.3)	31 (22.5)	26 (11.6)	14 (15.9)	9 (14.5)	22 (24.7)
College graduate	37 (46.2)	19 (34.5)	35 (25.4)	34 (15.2)	51 (58.0)	24 (38.7)	34 (38.2)
Income							
< $20k	20 (25.6)	11 (21.2)	33 (25.2)	77 (38.7)	25 (29.8)	13 (22.8)	47 (54.7)
$20k - < $75k	28 (35.9)	21 (40.4)	57 (43.5)	65 (32.7)	42 (50.0)	21 (36.8)	22 (25.6)
≥ $75k	12 (15.4)	2 (3.8)	16 (12.2)	15 (7.5)	8 (9.5)	5 (8.8)	3 (3.5)
Don’t know/Skipped	18 (23.1)	18 (34.6)	25 (19.1)	42 (21.1)	9 (10.7)	18 (31.6)	14 (16.3)
Health Insurance							
Private/Other	34 (42.5	25 (45.4)	46 (33.1)	57 (25.6)	18 (20.0)	11 (18.6)	10 (11.3)
Public	38 (47.5)	21 (38.2)	61 (43.9)	157 (70.4)	51 (56.7)	40 (67.8)	73 (82.0)
Uninsured	8 (10.0)	9 (16.4)	32 (23.2)	9 (4.0)	21 (23.3)	6 (13.6)	6 (6.7)
Last doctor’s visit							
< 1 year	68 (86.1)	43 (76.8)	103 (74.1)	180 (80.4)	64 (71.1)	57 (95.0)	66 (75.0)
≥ 1year/Never	11 (13.9)	13 (23.2)	36 (25.9)	44 (19.6)	26 (28.9)	3 (5.0)	22 (25.0)
Born in the US							
Yes	4 (5.0)	0 (0.0)	33 (23.9)	14 (6.3)	2 (2.2)	2 (3.2)	2 (2.3)
No	76 (95.0)	56 (100.0)	105 (76.1)	210 (93.7)	91 (97.8)	60 (96.8)	86 (97.7)
English spoken fluency							
Very well/Well	43 (54.4)	40 (74.1)	60 (46.2)	51 (24.8)	23 (26.4)	34 (58.6)	45 (53.6)
Not well/Not at all	36 (45.6)	14 (25.9)	70 (53.9)	155 (75.2)	64 (73.6)	24 (41.4)	39 (46.4)

*Abbreviations*: *SWANA *Southwest Asian and North African, *US *United States, *CHRNA *Cancer Community Health Resources and Needs Assessment

After adjustment for age group, South Asian (RR: 0.75, 95% CI: 0.58-0.98) and SWANA (RR: 0.70, 95% CI: 0.54-0.90) individuals were less likely to have received an up-to-date mammogram compared to the Afro-Caribbean group. Similar results were seen after adjustment for all variables (income, education, health insurance, check-up in the past year, nativity, and English language fluency) (Table 3).

### Breast cancer screening – CHS

In the CHS, n=1,244 women were eligible for breast cancer screening, answered mammogram screening questions, and identified as one of the included ethnic groups (Table 2). Up-to-date mammogram screening was lowest among South Asian individuals (64.2%), followed by Chinese (67.8%), Eastern European (68.9%), Latina (74.8%), and Afro-Caribbean (79.2%) individuals.

**Table 2:** Characteristics by racial/ethnic group, females ages 40–74 (CHS 2018, Crude), weighted %

	Eastern European (*n* = 101)	Afro-Caribbean (*n* = 304)	Latina (*n* = 633)	Chinese (*n* = 168)	South Asian (*n* = 38)
Mammogram < 2 years ago	68.9^a^	79.2	74.8	67.8^a^	64.2^a^
Age categories					
40–44	14.5	17.0	17.4	14.2	44.7
45–64	64.5	61.6	65.6	68.8	50.9
65–74	21.0	21.4	17.0	17.0	4.4
Education					
< High school	0.6	25.6	53.9	52.2	50.4
High school	24.5	23.6	17.8	22.0	23.4
Some college	19.5	24.3	15.9	9.4	6.6
College graduate	55.4	26.5	12.4	16.4	19.6
Poverty Rate					
<200%	33.1	48.3	73.5	76.7	72.8
200–399%	17.8	18.4	6.3	13.3	4.1
≥400%	49.1	33.3	20.2	13.0	23.1
Health Insurance					
Private/Other	60.0	48.6	27.5	26.6	23.6
Public	36.9	35.2	54.9	67.1	68.5
Uninsured	3.0	16.2	17.6	6.2	7.9
Has a regular provider					
Yes	92.5^b^	89.5^b^	86.8	91.0^b^	98.2^b^
No	7.5^a^	10.5^a^	13.2	9.0^a^	8.0^a^
Born in the US					
Yes	0.0^c^	18.2	24.7	1.4	0.0^c^
No	100.0^c^	81.8	75.3	98.6	100.0^c^

*Abbreviations*: *SWANA *Southwest Asian and North African, *US *United States, *CHS *New York City Community Health Survey

Weighted to the 2000 US population, only percentages are presented

^a^Estimate should be interpreted with caution. Estimate’s Relative Standard Error (a measure of estimate precision) is greater than 30%, or the 95% Confidence Interval half-width is greater than 10 or the sample size is too small, making the estimate potentially unreliable

^b^Compliment to the flagged estimate

^c^Estimate should be interpreted with caution. 95% Confidence Interval and Relative Standard Error are not calculated

^d^Data are suppressed due to imprecise and unreliable estimates

After adjustment for age group, there were no differences by ethnic group for up-to-date mammogram screening. Similar results were seen after adjustment for all other variables (poverty group, education, health insurance, having a regular provider, and nativity) (Table 3). A larger proportion of individuals age 40-44 was shown for South Asian (44.7%) women. Over half of Latina (53.9%), Chinese (52.2%), and South Asian (50.4%) women had <high school education, while over half of Eastern European women (55.4%) were college graduates. Poverty rate was highest (<200%) among Chinese (76.7%), Latina (73.5%) and South Asian (72.8%) women. Latina (17.6%) and Afro-Caribbean (16.2%) women were largely insured. Latina (13.2%) and Afro-Caribbean (10.5%) were least likely to have a regular provider. Greater than 80% of all groups were born outside the US, except for Latina women (75.3%).

**Table 3:** Adjusted relative risk of being up a mammogram <2 years ago among women ages 40-75, RR (95% CI)

	Cancer CHRNA 2021-2022		CHS 2018	
	Model 1 (n=746)	Model 2 (*n*=642)	Model 1 (*n*=1,250)	Model 2 (*n*=1,194)
Race or Ethnicity				
Eastern European	0.98 (0.80-1.20)	0.94 (0.75-1.17)	0.86 (0.69-1.08)	0.87 (0.67-1.12)
Afro-Caribbean	1 [Reference]	1 [Reference]	1 [Reference]	1 [Reference]
Latina	0.95 (0.79-1.15)	0.98 (0.78-1.23)	0.95 (0.85-1.05)	0.95 (0.85-1.06)
Chinese	0.95 (0.80-1.14)	0.88 (0.72-1.09)	0.85 (0.71-1.02)	0.87 (0.72-1.04)
Korean	0.85 (0.69-1.06)	0.94 (0.74-1.20)	—	—
South Asian	**0.75 (0.58-0.98)**	**0.74 (0.56-0.97)**	0.89 (0.59-1.34)	0.85 (0.57-1.28)
SWANA	**0.70 (0.54-0.90)**	**0.75 (0.57-0.98)**	—	—

*Abbreviations*: *SWANA *Southwest Asian and North African, *CHRNA *Cancer Community Health Resources and Needs Assessment, *CHS *New York City Community Health Survey, *RR *Relative risk, *CI *Confidence interval

Bolded values are significant at *p*<0.05

CHS data includes individuals aged 40-74

Model 1: adjusted for age group

Model 2: adjusted for age group, income or poverty group, education, health insurance, check-up <1 year ago for has a regular provider, nativity, and English spoken fluency (Cancer CHRNA only)

### Cervical cancer screening – Cancer CHRNA

In the Cancer CHRNA, n=1,117 women respondents were eligible for cervical cancer screening, answered Pap screening questions, and identified as one of the ethnic groups (Table 4). Up-to-date Pap screening was lowest among South Asian individuals (12.6%), followed by SWANA (35.9%), Korean (38.8%), Chinese (53.2%), Afro-Caribbean (55.6%), Eastern European (55.8%), and Latina (76.8%) individuals. A larger proportion of individuals age 45-64 was shown for Eastern European (53.7%), Korean (50.0%), SWANA (42.7%), and Chinese (40.0%) women. Over half of Korean (58.8%), South Asian (53.4%), and SWANA (51.3%) women were college graduates. Household income was lowest (<$20,000) among SWANA (43.7%) and Latina (30.6%) women. Korean (33.0%), Latina (25.0%), Afro-Caribbean (20.7%), South Asian (20.3%), and Eastern European (18.9%) women were largely insured. Korean (42.1%), Latina (31.6%), SWANA (31.0%), Eastern European (27.7%), and Afro-Caribbean (27.2%) were least likely to have visited a doctor in the past year. Greater than 80% of all groups were born outside of the US, except for South Asian (78.0%) and Latina women (67.2%). Speaking English not well or not at all was highest among Chinese (53.0%), Korean (51.4%), Afro-Caribbean (47.6%), and Latina (47.6%) women.

**Table 4:** Characteristics by racial/ethnic group, females ages 21-65 (Cancer CHRNA 2021-2022), n (%)

	Eastern European (*n*=95)	Afro-Caribbean (*n*=115)	Latina (*n*=263)	Chinese (*n*=250)	Korean (*n*=116)	South Asian (*n*=161)	SWANA (*n*=117)
Pap screening <3 years ago	53 (55.8)	64 (55.6)	202 (76.8)	133 (53.2)	45 (38.8)	78 (12.6)	42 (35.9)
Age, categories							
21-24	8 (8.4)	17 (14.8)	27 (10.3)	16 (6.4)	8 (6.9)	30 (18.6)	12 (10.3)
25-44	36 (37.9)	59 (51.3)	140 (53.2)	134 (53.6)	50 (43.1)	87 (54.0)	55 (47.0)
45-64	51 (53.7)	39 (33.9)	96 (36.5)	100 (40.0)	58 (50.0)	44 (27.3)	50 (42.7)
Education							
<High school	5 (5.3)	11 (9.7)	76 (29.2)	78 (31.2)	1 (0.9)	24 (14.9)	15 (12.8)
High school	14 (14.7)	14 (12.4)	47 (18.1)	60 (24.0)	22 (19.3)	20 (12.4)	7 (6.0)
Some college	30 (31.6)	32 (28.3)	66 (25.4)	41 (16.4)	24 (21.0)	31 (19.3)	35 (29.9)
College graduate	46 (48.2)	56 (49.6)	71 (27.3)	71 (28.4)	67 (58.8)	86 (53.4)	60 (51.3)
Household Income							
< $20k	21 (22.6)	15 (14.3)	76 (30.6)	49 (20.9)	24 (22.9)	39 (26.0)	49 (43.7)
$20k - < $75k	41 (44.1)	46 (43.8)	112 (45.2)	105 (44.9)	52 (49.5)	58 (38.7)	44 (39.3)
$75k	16 (17.2)	6 (5.7)	26 (10.5)	29 (12.4)	20 (19.0)	17 (11.3)	6 (5.4)
Not reported	15 (16.1)	38 (36.2)	34 (13.7)	51 (21.8)	9 (8.6)	36 (24.0)	13 (11.6)
Health Insurance							
Private/Other	41 (43.2)	39 (35.1)	83 (31.9)	101 (41.2)	40 (34.8)	37 (24.2)	23 (20.0)
Public	36 (37.9)	49 (44.1)	112 (43.1)	121 (49.4)	37 (32.2)	85 (55.6)	80 (69.6)
Uninsured	18 (18.9)	23 (20.7)	65 (25.0)	23 (9.4)	38 (33.0)	31 (20.3)	12 (10.4)
Last doctor’s visit							
< 1 year	68 (72.3)	83 (72.8)	180 (68.4)	185 (74.9)	66 (57.9)	124 (79.0)	80 (69.0)
1 year/Never	26 (27.7)	31 (27.2)	83 (31.6)	62 (25.1)	48 (42.1)	33 (21.0)	36 (31.0)
Born in the US							
Yes	12 (12.8)	19 (16.5)	86 (32.8)	34 (13.7)	19 (16.5)	35 (22.0)	23 (18.8)
No	82 (87.2)	96 (83.5)	176 (67.2)	215 (86.3)	96 (83.5)	124 (78.0)	93 (80.2)
English spoken fluency							
Very well/Well	62 (66.0)	89 (80.2)	131 (52.4)	110 (47.0)	53 (48.6)	113 (73.4)	82 (73.9)
Not well/Not at all	32 (34.0)	22 (47.6)	119 (47.6)	124 (53.0)	56 (51.4)	41 (26.6)	29 (26.1)

*Abbreviations*: *SWANA *Southwest Asian and North African, *US *United States, *CHRNA *Cancer Community Health Resources and Needs Assessment

After adjustment for age group, Eastern European (RR: 0.71, 95% CI: 0.58-0.85), Afro-Caribbean (RR: 0.74, 95% CI: 0.62-0.88), Chinese (RR: 0.68, 95% CI: 0.60-.78), Korean (RR: 0.49, 95% CI: 0.39-0.62), South Asian (RR: 0.66, 95% CI: 0.56-0.78) and SWANA (RR: 0.47, 95% CI: 0.36-0.60) individuals were less likely to have received an up-to-date Pap test compared to Latina individuals. Similar results were seen after adjustment for all variables (age group, income, education, health insurance, check-up in the past year, nativity, and English language fluency) (Table 6).

### Cervical cancer screening – CHS

In the CHS, n=1,983 women were eligible for cervical cancer screening, answered Pap screening questions, and identified as one of the ethnic groups (Table 5). Up-to-date Pap screening was lowest among South Asian individuals (51.5%), followed by Chinese (57.9%), Eastern European (78.7%), Latina (80.5%), and Afro-Caribbean (84.9%) individuals. A large proportion of individuals age 45-64 was shown for Afro-Caribbean (53.0%) women. Over half of Eastern European women (77.5%) women were college graduates. Poverty rate was highest (<200%) among Chinese (67.6%), Latina (65.9%) and South Asian (45.8%) women. Latina (21.8%) and South Asian (12.1%) women were largely insured. Latina (21.2%) and Eastern European (13.3%) were least likely to have a regular provider. Greater than 94% of all groups were born outside the US, except for Latina women (63.9%).

**Table 5:** Characteristics by racial/ethnic group, females ages 21-64 (CHS 2017, crude), weighted %

	Eastern European (*n*=104)	Afro-Caribbean (*n*=272)	Latina (*n*=1,269)	Chinese (*n*=295)	South Asian (*n*=43)
Pap screening <3 years ago	78.3	84.9	80.5	57.9	51.5^a^
Age categories					
21-24	^d^	8.7^a^	7.1	6.1^a^	^d^
25-44	52.3^a^	38.3	52.2	56.5	67.4^a^
45-64	44.4^a^	53.0	40.7	37.4	24.1^a^
Education					
<High school	0.0^c^	7.4	38.3	26.5	4.3
High school	14.4^a^	27.3	20.3	34.7	29.7
Some college	8.1^a^	30.5	23.3	11.1	26.7
College graduate	77.5^a^	34.8	18.1	27.7	39.3
Poverty Rate					
<200%	38.7	43.0	65.9	67.6	45.8
200-399%	13.4	27.9	17.9	14.2	18.8
400%	47.9	29.1	16.2	18.2	35.4
Health Insurance					
Private/Other	66.6	54.7	29.1	38.2	60.2
Public	23.6	36.3	49.1	54.0	27.7
Uninsured	9.8	9.0	21.8	7.8	12.1
Has a regular provider					
Yes	86.7^a^	94.4^a^	78.9	90.5	88.7^a^
No	13.3^a^	5.6^a^	21.1	9.5	11.3^a^
Born in the US					
Yes	0.0^c^	0.0^c^	36.1	5.2	0.0^c^
No	100.0^c^	100.0^c^	63.9	94.8	100.0^c^

*Abbreviations*: *SWANA *Southwest Asian and North African, *US *United States, *CHS *New York City Community Health Survey Weighted to the 2000 US population, only percentages are presented

^a^Estimate should be interpreted with caution. Estimate’s Relative Standard Error (a measure of estimate precision) is greater than 30%, or the 95% Confidence Interval half-width is greater than 10 or the sample size is too small, making the estimate potentially unreliable

^b^Compliment to the flagged estimate

^c^Estimate should be interpreted with caution. 95% Confidence Interval and Relative Standard Error are not calculated

^d^Data are suppressed due to imprecise and unreliable estimates

After adjustment for age group, Chinese (RR: 0.71, 95% CI: 0.63-0.81) and South Asian (RR: 0.64, 95% CI: 0.43-0.95) individuals were less likely to have received an up-to-date Pap test compared to Latina individuals. Similar results were seen after adjustment for all variables (poverty group, education, health insurance, having a regular provider, and nativity) (Table 6).

**Table 6:** Adjusted relative risk of a Pap test <3 years ago among women ages 21-65, RR (95% CI)

	Cancer CHRNA 2021-2022		CHS 2017	
	Model 1 (*n*=1,117)	Model 2 (*n*=986)	Model 1 (*n*=1,983)	Model 2 (*n*=1,934)
Race or Ethnicity				
Eastern European	**0.71 (0.58-0.85)**	**0.63 (0.53-0.76)**	0.97 (0.86-1.09)	0.93 (0.80-1.08)
Afro-Caribbean	**0.74 (0.62-0.88)**	**0.68 (0.55-0.83)**	1.06 (0.98-1.14)	1.04 (0.95-1.30)
Latina	1 [Reference]	1 [Reference]	1 [Reference]	1 [Reference]
Chinese	**0.68 (0.60-0.78)**	**0.61 (0.53-0.71)**	**0.71 (0.63-0.81)**	**0.67 (0.57-0.77)**
Korean	**0.49 (0.39-0.62)**	**0.46 (0.35-0.59)**	—	—
South Asian	**0.66 (0.56-0.78)**	**0.59 (0.49-0.72)**	**0.64 (0.43-0.95)**	**0.59 (0.38-0.90)**
SWANA	**0.47 (0.36-0.60)**	**0.48 (0.37-0.62)**	—	—

*Abbreviations*: *SWANA *Southwest Asian and North African, *CHRNA *Cancer Community Health Resources and Needs Assessment, *CHS *New York City Community Health Survey, *RR *Relative risk, *CI *Confidence interval

Bolded values are significant at *p*<0.05

CHS data includes individuals aged 21-64

Model 1: adjusted for age group

Model 2: adjusted for age group, income or poverty group, education, health insurance, check-up <1 year ago for has a regular provider, nativity, and English spoken fluency (Cancer CHRNA only)

### CRC screening – Cancer CHRNA

In the Cancer CHRNA, n=790 respondents were eligible for CRC screening, answered CRC screening questions, and identified as one of the ethnic groups (Table 7). Up-to-date colonoscopy screening was lowest among South Asian individuals (26.8%), followed by Afro-Caribbean (34.6%), Latine (37.3%), Korean (39.4%), SWANA (42.0%), Chinese (58.3%), and Eastern European (69.0%) individuals. Over half of Chinese individuals (61.9%) had <high school education, while over half of South Asian individuals (58.5%) were college graduates. Household income was lowest (<$20,000) among SWANA (64.3%) and Chinese (49.3%) women. Latine (23.6%) and Korean (15.6%) individuals were largely insured. Korean (42.1%), Latine (27.5%), Afro-Caribbean (26.9%), SWANA (24.8%), and Korean (23.5%) individuals were least likely to have visited a doctor in the past year. Greater than 94% of all groups were born outside of the US, except for Latine individuals (75.4%). Speaking English not well or not at all was highest among Chinese (84.3%), Korean (82.6%), Latine (56.9%), and Eastern European (52.9%) individuals.

**Table 7:** Characteristics by racial/ethnic group, ages 50-75 (Cancer CHRNA 2021-2022), n (%)

	Eastern European (*n*=87)	Afro-Caribbean (*n*=52)	Latine (*n*=110)	Chinese (*n*=252)	Korean (*n*=99)	South Asian (*n*=71)	SWANA (*n*=119)
Any CRC screening	66 (75.9)	24 (46.2)	50 (45.5)	163 (64.7)	46 (46.5)	25 (35.2)	56 (47.1)
Colonoscopy <10 years ago	60 (69.0)	18 (34.6)	41 (37.3)	147 (58.3)	39 (39.4)	19 (26.8)	50 (42.0)
Sigmoidoscopy <5 years ago	1 (1.2)	4 (7.7)	0 (0.0)	2 (0.8)	5 (5.1)	0 (0.0)	3. (2.5)
FOBT <1 year ago	11 (12.6)	5 (9.6)	16 (14.7)	41 (16.3)	2 (2.1)	9 (13.0)	6 (5.1)
Age, categories							
50-64	51 (58.6)	38 (73.1)	87 (79.1)	102 (40.5)	60 (60.6)	60 (84.5)	69 (58.0)
65-75	36 (41.4)	14 (26.9)	23 (20.9)	150 (59.5)	39 (39.4)	11 (15.5)	50 (42.0)
Sex							
Male	28 (32.2)	13 (25.5)	34 (30.9)	84 (33.3)	29 (29.3)	32 (45.1)	56 (48.3)
Female	59 (67.8)	39 (74.5)	76 (69.1)	168 (66.7)	70 (70.7)	39 (54.9)	60 (51.7)
Education							
<High school	6 (6.9)	11 (21.6)	43 (40.2)	154 (61.9)	3 (3.1)	16 (22.5)	36 (30.2)
High school	15 (17.2)	14 (27.4)	17 (15.9)	46 (18.5)	18 (19.2)	14 (19.7)	16 (13.5)
Some college	28 (32.2)	15 (29.4)	20 (18.7)	24 (9.6)	18 (19.2)	10 (14.1)	23 (19.3)
College graduate	38 (43.7)	11 (21.6)	27 (25.2)	25 (10.0)	55 (58.5)	31 (43.7)	44 (37.0)
Household Income							
< $20k	26 (30.2)	11 (22.0)	33 (31.7)	112 (49.3)	29 (31.8)	15 (22.4)	74 (64.3)
$20k - < $75k	31 (36.1)	17 (34.0)	36 (34.6)	48 (21.1)	47 (51.7)	25 (37.3)	23 (20.0)
$75k	14 (16.3)	1 (2.0)	12 (11.5)	13 (5.7)	6 (6.6)	7 (10.4)	1 (0.9)
Not reported	15 (17.4)	21 (42.0)	23 (22.1)	54 (23.8)	9 (9.9)	20 (29.9)	17 (14.8)
Health Insurance							
Private/Other	35 (41.2)	28 (54.9)	36 (32.7)	46 (18.6)	21 (21.9)	9 (13.0)	13 (11.1)
Public	43 (50.6)	18 (35.3)	48 (43.7)	195 (78.6)	60 (62.5)	52 (75.4)	96 (82.1)
Uninsured	7 (8.2)	5 (9.8)	26 (23.6)	7 (2.8)	15 (15.6)	8 (11.6)	8 (6.8)
Last doctor’s visit							
< 1 year	71 (83.5)	38 (73.1)	79 (72.5)	202 (81.8)	75 (76.5)	66 (94.3)	88 (75.2)
1 year/Never	14 (16.5)	14 (26.9)	30 (27.5)	45 (18.2)	23 (23.5)	4 (5.7)	29 (24.8)
Born in the US							
Yes	5 (5.7)	0 (0.0)	27 (24.6)	12 (4.8)	1 (1.0)	1 (1.4)	2 (1.7)
No	82 (94.3)	51 (100.0)	83 (75.4)	237 (95.2)	98 (99.0)	69 (98.6)	114 (98.3)
English spoken fluency							
Very well/Well	41 (47.1)	34 (68.0)	44 (43.1)	37 (15.7)	16 (17.4)	41 (60.3)	59 (52.7)
Not well/Not at all	46 (52.9)	16 (32.0)	58 (56.9)	198 (84.3)	76 (82.6)	27 (39.7)	53 (47.3)

*Abbreviations*: *SWANA *Southwest Asian and North African, *US *United States, *CHRNA *Cancer Community Health Resources and Needs Assessment, *CRC *Colorectal cancer screening, *FOBT *Fecal occult blood test

After adjustment for age group, Afro-Caribbean (RR: 0.50, 95% CI: 0.33-0.75), Latine (RR: 0.57, 95% CI: 0.44-0.76), Chinese (RR: 0.80, 95% CI: 0.68-0.96), Korean (RR: 0.57, 95% CI: 0.44-0.75), South Asian (RR: 0.43, 95% CI: 0.28-0.65) and SWANA (RR: 0.63, 95% CI: 0.49-0.81) individuals were less likely to have received an up-to-date colonoscopy compared to Eastern European individuals. Similar results were seen after adjustment for all variables (sex, income, education, health insurance, check-up in the past year, nativity, and English language fluency), but Chinese individuals were no longer significantly different from the Eastern European group (Table 9). A separate model was run using the Cancer CHRNA data, predicting up to date CRC screening using colonoscopy, sigmoidoscopy, and FOBT recommendations; similar results were shown to colonoscopy screening; all groups but Chinese were significant in adjusted analyses (Additional file 3).

In the CHS, *n*=4,682 respondents were eligible for CRC screening, answered CRC screening questions, and identified as one of the ethnic groups (Table 8). Up-to-date colonoscopy screening was lowest among SWANA individuals (53.3%), followed by South Asian (34.6%), Chinese (62.8%), Eastern European (68.2%), Latine (71.1%), and Afro-Caribbean (72.4%) individuals. Over half of Chinese (53.7%) and Latine (50.2%) individuals had <high school education, while over half of Eastern European (55.6%) and SWANA (53.5%) individuals were college graduates. Poverty rate was highest (<200%) among Chinese (71.2%), Latine (69.7%) and South Asian (64.8%) individuals. Latine (17.5%), South Asian (13.8%), and Afro-Caribbean (12.6%) individuals were largely insured. SWANA (22.8%), Latine (15.1%), and Eastern European (14.3%) individuals were least likely to have a regular provider. Greater than 88% of all groups were born outside the US, except for Latine individuals (69.0%).

**Table 8:** Characteristics by racial/ethnic group, ages 50-74 (CHS 2018-2020, crude), weighted percent

	Eastern European (*n* = 378)	Afro-Caribbean (*n* = 968)	Latine (*n* = 2,389)	Chinese (*n* = 642)	South Asian (*n* = 204)	SWANA (*n* = 101)
Colonoscopy < 10 years ago	68.2	72.4	71.1	62.8	58.2a	53.3^a^
Age categories						
50–64	60.4	71.9	76.0	68.2	87.4	71.8
65–74	39.6	28.1	24.0	31.8	12.6	28.2
Sex						
Male	40.5	39.2	38.8	44.8	68.2^a^	67.8^a^
Female	59.5	60.8	61.2	55.2	31.8^a^	32.2^a^
Education						
< High school	8.5^a^	23.8	50.2	53.7	24.5^a^	6.8^a^
High school	16.7	27.5	20.2	30.6	26.2^a^	19.6^a^
Some college	19.2	23.0	18.8	5.7	10.3^a^	20.1
College graduate	55.6	25.7	10.8	10.0	39.0^a^	53.5^a^
Poverty Rate						
<200%	41.4	46.7	69.7	71.2	64.8^a^	33.2^a^
200–399%	24.2	20.0	13.3	14.5	15.7	19.4
≥400%	34.4	33.4	17.0	14.3	19.5	47.4^a^
Health Insurance						
Private/Other	45.3	49.2	28.9	31.6	31.1	57.8
Public	47.3	38.2	53.6	66.7	55.1	34.9
Uninsured	7.4	12.6	17.5	1.7	13.8	7.3
Has a regular provider						
Yes	85.7	87.8	84.9	97.3	94.8^b^	77.2^a^
No	14.3	12.2	15.1	2.7	5.2^a^	22.8^b^
Born in the US						
Yes	0.0^c^	11.1	31.0	3.1^a^	0.0^c^	5.8
No	100.0^c^	88.9	69.0	96.9^b^	100.0^c^	94.2

*Abbreviations*: *SWANA *Southwest Asian and North African, *US *United States, *CHS *New York City Community Health Survey

Weighted to the 2000 US population, only percentages are presented

^a^Estimate should be interpreted with caution. Estimate’s Relative Standard Error (a measure of estimate precision) is greater than 30%, or the 95% Confidence Interval half-width is greater than 10 or the sample size is too small, making the estimate potentially unreliable

^b^Compliment to the flagged estimate

^c^Estimate should be interpreted with caution. 95% Confidence Interval and Relative Standard Error are not calculated

After adjustment for age group, Chinese (RR: 0.86, 95% CI: 0.76-0.98) and SWANA (RR: 0.76, 95% CI: 0.59-0.97) individuals were less likely to have received and up-to-date colonoscopy compared to Afro-Caribbean individuals. Similar results were seen after adjustment for all variables (sex, poverty group, education, health insurance, having a regular health provider, and nativity) (Table 9).

**Table 9:** Adjusted relative risk of a colonoscopy screening <10 years ago among individuals ages 50-75, RR (95% CI)

	Cancer CHRNA 2021-2022		CHS – 2017-2020	
	Model 1 (*n*=786)	Model 2 (*n*=684)	Model 1 (*n*=4,663)	Model 2 (*n*=4,446)
Race or Ethnicity				
Eastern European	1 [Reference]	1 [Reference]	0.92 (0.80-1.06)	0.92 (0.79-1.07)
Afro-Caribbean	**0.50 (0.33-0.75)**	**0.50 (0.32-0.79)**	1 [Reference]	1 [Reference]
Latine	**0.57 (0.44-0.76)**	**0.74 (0.56-0.98)**	0.99 (0.91-1.08)	1.03 (0.93-1.13)
Chinese	**0.80 (0.68-0.96)**	0.88 (0.72-1.08)	**0.86 (0.76-0.98)**	**0.87 (0.76-1.00)**
Korean	**0.57 (0.44-0.75)**	**0.68 (0.51-0.91)**	**—**	**—**
South Asian	**0.43 (0.28-0.65)**	**0.46 (0.30-0.69)**	0.86 (0.71-1.05)	0.84 (0.68-1.58)
SWANA	**0.63 (0.49-0.81)**	**0.69 (0.52-0.91)**	**0.76 (0.59-0.97)**	**0.73 (0.55-0.97)**

*Abbreviations*: *SWANA *Southwest Asian and North African, *CHRNA *Cancer Community Health Resources and Needs Assessment, *CHS *New York City Community Health Survey, *RR *Relative risk, *CI *Confidence interval

Bolded values are significant at p<0.05

CHS data includes individuals aged 50-74

Model 1: adjusted for age group and sex

Model 2: adjusted for age group, sex, income or poverty group, education, health insurance, check-up <1 year ago for has a regular provider, nativity, and English spoken fluency (Cancer CHRNA only)

## Discussion

This study examined breast, cervical, and CRC screening among seven diverse racial and ethnic groups from two recent NYC datasets. In this updated examination of cancer screening rates, we found low up-to-date cancer screening prevalence for many non-White groups, especially in the more community focused Cancer CHRNA sample.

The Healthy People 2030 target for breast cancer screening (receiving an a mammogram in the past 2 years among women aged 50-74 years), is 80.3%; this age grouping differs from our data reporting (age 40-75 years) [34]. No groups met this target for breast cancer screening. In the Cancer CHRNA, an up-to-date mammogram was especially low for SWANA (50.6%), South Asian (54.8%), Korean (63.4%), Latina (69.0%), and Chinese (69.6%) women. In the CHS, an up-to-date mammogram was especially low for South Asian (64.2%), Chinese (67.8%), and Eastern European (68.9%).

Similarly, the 2022 NY BRFSS found low mammogram screening prevalence among NH Asian and PI (69.3%) and NH White (73.1%) women [13]. National Health Interview Survey (NHIS) data has also found lower mammogram screening rates among NH Asian American women and recent immigrants [35–37]

The Healthy People 2030 target for cervical cancer screening (receiving a Pap test in the past 3 years among women aged 21-65 years) is 79.2% [38]. No groups in the Cancer CHRNA met this target, and only the Latina (80.5%) and Afro-Caribbean (84.9%) groups in the CHS met this target. An up-to-date Pap test was especially low in the Cancer CHRNA among South Asian (12.6%), SWANA (35.9%), Korean (53.2%), Chinese (53.2%), Afro-Caribbean (55.5%), and Eastern European (55.8%) women. An up-to-date Pap test was especially low in the CHS among South Asian (51.5%) and Chinese (57.9%) women.

Similarly, the 2020 NY BRFSS found low cervical cancer screening among NH Asian and PI women (63.6%) [12]. NHIS data has also found lower cervical cancer screening rates among NH Asian women and recent immigrants; data from 2018 found that the weighted cervical cancer screening rate was 70.3% among NH Asian women, compared to 81.1% among NH White women, while data from 2008, 2010, and 2013 found that the weighted cervical cancer screening rate was 59.9% among Asian women who had lived in the US for <10 years [35–37]. Our cervical cancer screening rates were lower for many subgroups (12.6% among South Asian, 38.8% among Korean, and 53.2% among Chinese women in the Cancer CHRNA, and 51.5% among South Asian and 57.9% among Chinese women in the CHS.

The Healthy People 2030 target for CRC screening (receiving a CRC screening in the recommended time frame for adults aged 45-75 years) is 72.8% [39]. We examine colonoscopy only using individuals 50-75 years. No groups met this target using colonoscopy screening, and only the Eastern European group in the Cancer CHRNA met this target for any CRC screening modality (75.9%). In the Cancer CHRNA, and up-to-date colonoscopy screening was especially low among South Asian (26.8%), Afro-Caribbean (34.6%), Latine (37.3%), SWANA (42.0%), and Chinese (58.3%) individuals. In the CHS, an up-to-date colonoscopy screening was especially low among South Asian (58.2%), SWANA (53.3%), and Chinese (62.8%) individuals. Similarly, 2022 NY BRFSS found low CRC screening among individuals aged 45-75 years among NH Asian and PI (49.8%) and Latine (64.7%) groups [10].

The DOHMH has focused efforts to increase colonoscopy screening among minoritized groups in NYC over the past two decades, especially among Black and Latine groups [7]. While the Asian American group experienced an overall increase in colonoscopy screening (24% in 2003 to 65% in 2018 among Asians and Pacific Islanders age 50 and older), prevalence has remained low among the South Asian subgroup; using combined 2014-2018 CHS data, 61.2% of South Asians age ≥50 have received a colonoscopy in the past 10 years, compared to 68.6% of NH Whites, 70.2% of NH Blacks, and 71.3% of Hispanics [8]. Our analyses indicate low CRC screening prevalence in other minoritized groups lacking representation in local and national datasets, including SWANA, Eastern European, and Afro-Caribbean, as well as disaggregated Asian American groups (South Asian, Korean, and Chinese).

Barriers to cancer screenings among racial and ethnic minoritized groups include cost, time, lack of provider recommendations, limited knowledge, and a shortage of campaigns or educational materials in non-English languages. Other challenges may include limitations in healthcare and language access, as well as limited resources to reach and engage immigrant communities [5, 40, 41]. A NYC study examining Afro-Caribbean groups found misconceptions about CRC, embarrassment, and cost of healthcare to be barriers [42, 43]. A NYC focus group study on Russian-speaking immigrants found potential barriers to include individual attitudes and behaviors, cultural beliefs, and structural issues [44]. A qualitative study on South Asian women in New Jersey found low perceived breast cancer risk and cultural and structural barriers to screening, such as deprioritizing women’s health and family responsibilities [45], while a study among South Asian and African/African American Muslim women in NYC found that language barriers, gender roles, and lack of knowledge about preventive care needed to be addressed [46]. There may be gaps and systematic challenges to reaching and engaging these populations, with a need to factor cultural norms, values, and religion (e.g., modesty, religious values) in health promotion and screening messaging [46].

More culturally and community tailored interventions and information dissemination are needed for groups with low screening uptake [40, 41, 46, 47], as these types of interventions can help increase participation in cancer and reduce race and ethnicity inequalities in access to screening programs [48]. A systemic review found that screening rates for colorectal, breast, and cervical cancer were higher among patients that were provided navigation services, among populations disproportionately affected by health care disparities, and services such as lay health workers, language translation, and reminder calls were the most important aspects of tailoring [49]. In NYC, a social marketing, lay health worker intervention improved breast and cervical cancer screening uptake among Muslim women [50]. In Seattle, Washington, a culturally tailored, promotor(a)-led home health intervention increased CRC screening among a rural community of Hispanic individuals [51].

Sampling modalities and populations represented may also help explain some of the findings in screenings, as well as differences across datasets; the Cancer CHRNA, which saw lower prevalence among many groups in comparison to the CHS, sampled more in non-English speaking groups; but both surveys included Chinese, Spanish, Haitian Creole, Russian, and Bengali languages. Bengali and Haitian Creole were added to the CHS in 2018, thus would not be applicable to Pap test which collected data for 2017 only. The Cancer CHRNA also included Urdu and Korean languages. The top languages spoken by foreign-born individuals with limited English proficiency in NYC include many of the languages surveyed: Chinese, Russian, Bengali, Haitian Creole, Arabic, and Korean [52, 53]. The Cancer CHRNA was conducted directly in minoritized and immigrant communities with the help of CHWs, while the CHS was phone-administered and can exclude individuals who are not able to be reached by phone. Additionally, CHS data was collected prior to the COVID-19 pandemic for mammogram and Pap testing (colonoscopy screening includes data from 2018-2020), while the Cancer CHRNA was collected between 2021-2022; lack of access to care during COVID-19 may explain some of the lower screening rates in the Cancer CHRNA.

Some limitations should be noted. First, survey data were self-reported, and we cannot distinguish between screening and diagnostic tests. Second, Central Asian, Indo-Caribbean, detailed Latine groups, and mixed race were excluded due to sample size, and other groups were excluded once subset for age and sex (e.g., subgroups of Korean and SWANA women aged 40-75 in one year of CHS data for mammogram screening); once subset for age and sex, some groups were small and should interpreted with caution (e.g., Afro-Caribbeans, Korean, and South Asian males in the Cancer CHRNA), as well as flagged estimates from the CHS. Additionally, this data may not be generalizable to the NYC population, as the subgroups chosen were based off the oversampling for the Cancer CHRNA survey. Third, the CHS only collects colonoscopy modality for CRC screening; many average risk individuals choose to have FOBT testing. Fourth, different age cut-offs were used for the CHS due to the DOHMH coded age categories (74 vs. 75, and 64 vs. 65 years). Fifth, screening guidelines are continually updated; for example, CRC screening is now recommended beginning at age 45 (previously 50 years) [29, 54], and mammogram screening age initiation (40 or 50 years) has differed over time and by recommendation [25, 55]. CHS colonoscopy screening was collected in 2018-2020, prior to new guidelines; thus, the decision was made to include age 50-75 years for analyses. Finally, there is the potential for an overlap in individuals taking the survey, but we are not able to identify participants in the CHS dataset. Given the different recruitment methods and languages used for the survey, we do not expect any overlap to be significant.

## Conclusion

We found large variations in screening rates by detailed racial and ethnic groups in NYC. This highlights the importance of data disaggregation, which helps to identify emerging disparities when granular racial and ethnic categories are available. Continued efforts are needed to investigate barriers to cancer screening in these diverse, racial and ethnic minoritized groups in NYC, as well as to increase knowledge and screening efforts using culturally and linguistically tailored strategies and interventions. Evidence-based, culturally tailored interventions utilizing CHWs have the potential to increase screening rates among groups susceptible to social determinants of health and systems-level challenges to accessing the healthcare system [50, 56]. The Community Preventive Services Task Force strongly recommends patient navigation services and interventions engaging CHWs for breast, cervical, and CRC screening [57]. Future researchers should consider these findings when conducting surveys and interventions on cancer screenings in order to reach and engage minoritized communities. Information and messages tailored to cultural- and community-level norms and values should be included in order to broaden reach, and disaggregation of racial and ethnic groups should be conducted at whatever extent possible.

## Supplementary Information

Supplementary Material 1.

Supplementary Material 2.

Supplementary Material 3.

## Data Availability

Cancer CHRNA data is available on request from the corresponding author upon reasonable request. CHS data is available through a data use agreement with the NYC DOHMH.
