# Supplementary material for: Prevalence of breast, cervical, and colorectal cancer screenings among select New York City populations
Source: BMC Cancer. 2025 Sep 30;25:1469. doi: 10.1186/s12885-025-14763-z (PMC12482105; doi:10.1186/s12885-025-14763-z)
Supplement: Supplementary file 1 — Supplementary Material 1. [file 12885_2025_14763_MOESM1_ESM.docx]

Additional file 1

**Screening questions:**

1. What is your age?
2. What is the zip code where you live currently (BRFSS)?

**Write-in:** ______________ Don’t Know/Not Sure

# Self-Reported Health Status

1. Would you say that in general your health is…

- Excellent
- Very Good
- Good
- Fair
- Poor
- Don’t Know/Not Sure
- Decline to State

# Health care access

1. About how long has it been since you last visited a doctor for a routine checkup? (BRFSS for q/ NYC CHS/ regional chrna)

- Never
- Less than 1 year ago
- 1 year ago, but less than 2 years ago
- 2 years ago, but less than 5 years ago
- 5 or more years ago
- Don’t Know/Not Sure
- Decline to State

# Financial barriers to health care

1. In the past 12 months was there a time when you needed to see a doctor, but could not because of cost? (BRFSS)

- Yes
- No
- Don’t know/Not sure

Physical Activity (OCHIN)

1. On average, how many days per week do you engage in moderate to strenuous exercise such as running, calisthenics, golf, gardening, or walking for exercise?? (0-7)

Numeric answer; Days:________

- Don’t Know/ Not sure

1. On average, how many minutes do you engage in exercise at this level? (0-90)

Numeric answer; Minutes:______

- Other amount
- Don’t Know/ Not sure

# Diet (DSQ)

1. During the past month, how often did you drink 100% pure fruit juices? Do not include fruit-flavored drinks with added sugar or fruit juice you made at home and added sugar to. Examples of 100% pure fruit juices: apple, orange, mango, grape, grapefruit, guava, jallab, qamar-al-deen, strawberry, apricot, mors (mixed berry juice), maracuya/chinola juice, etc.? Do not include fruit-flavored drinks with added sugar or fruit juice you made at home and added sugar to**.**

- Never
- 1 time last month
- 2-3 times last month
- 1 time per week
- 2 times per week
- 3-4 times per week
- 5-6 times per week
- 1 time per day
- 2-3 times per day
- 4-5 times per day
- 6 or more times per day

1. During the past month, how often did you eat a green leafy or lettuce salad, with or without other vegetables?

- Never
- 1 time last month
- 2-3 times last month
- 1 time per week
- 2 times per week
- 3-4 times per week
- 5-6 times per week
- 1 time per day
- 2 or more times per day

1. During the past month, how often did you eat brown rice or other cooked whole grains? Do not include white rice. Examples of cooked whole grains: whole wheat noodles, bajra, freekeh, couscous, quipe, quinoa, buckwheat (grechka), millet (pshenka) or pearl barley (perlovka).

- Never
- 1 time last month
- 2-3 times last month
- 1 time per week
- 2 times per week
- 3-4 times per week
- 5-6 times per week
- 1 time per day
- 2 or more times per day

1. During the last month, how often did you eat red meat, such as beef, pork, or ox? Do not include chicken, turkey or seafood. Include red meat you had in sandwiches, lasagna, stew, and other mixtures. Red meats may also include veal, lamb, and any lunch meats made with these meats.

- Never
- 1 time last month
- 2-3 times last month
- 1 time per week
- 2 times per week
- 3-4 times per week
- 5-6 times per week
- 1 time per day
- 2 or more times per day

1. During the last month, how often did you eat any processed meat, such as bacon, lunch meats, or hot dogs? Include processed meats you had in sandwiches, soups, pizza, casseroles, and other foods. Processed meats are those preserved by smoking, curing, or salting, or by the addition of preservatives. Examples are: ham, bacon, pastrami, salami, sausages, hot dogs, salchichon, chorizo, salo, basturma, kolbasa or spam.

- Never
- 1 time last month
- 2-3 times last month
- 1 time per week
- 2 times per week
- 3-4 times per week
- 5-6 times per week
- 1 time per day
- 2 or more times per day

Food Security (OCHIN)

1. (I/We) worried whether (my/our) food would run out before (I/we) got money to buy more in the last 12 months?

- Often true
- Sometimes true
- Never true
- Decline to State

1. The food that (I/we) bought just didn’t last, and (I/we) didn’t have money to get more in the last 12 months?

- Often true
- Sometimes true
- Never true
- Decline to State

# Sociodemographic measures

1. What is your age? (HINTS)

**Write in:**______Years old (18–110)

1. What terms best express how you describe your gender identity? (Check all that apply) (CHS)

- Man
- Woman
- Transgender man
- Transgender women
- Non-binary person
- Other: A gender not mentioned
- Don’t Know/Not Sure
- Prefer not to answer ______________________________

1. What was your sex assigned at birth? (CHS)

- Male
- Female
- Don’t Know/Not Sure
- Perfer not to answer

1. Do you currently rent or own your home? (HINTS)

- Own
- Rent
- Occupied without paying monetary rent

1. Which of these comes closest to your own feelings about your household's income these days? (Gallup)

- Living comfortably on present income
- Getting by on present income
- Finding it difficult on present income
- Finding it very difficult on present income

1. Do you have any kind of health care coverage, including health insurance, prepaid plans such as HMOs, or government plans such as Medicare, Medicaid or Indian Health Service? (BRFSS)

- Yes
- No
- Don’t Know/Not Sure
- Decline to State

1. What is the primary source of your health care coverage? (BRFSS)

- A plan purchased through an employer or union (including plans purchased through another person's employer)
- A plan that you or another family member buys on your own
- Medicare
- Medicaid or other state program
- TRICARE (formerly CHAMPUS), VA, or Military
- Alaska Native, Indian Health Service, Tribal Health Services
- Some other source (please specify): ________________________
- Don’t Know/Not Sure

1. What is the highest grade or level of schooling you completed? (HINTS)

- Less than 8 years
- 8 through 11 years
- 12 years or completed high school
- Post high school training other than college (vocational or technical)
- Some college
- College graduate
- Postgraduate

1. What is your marital status? Mark only one. (HINTS/BRFSS)

- Married
- Living as married
- Divorced
- Widowed
- Separated
- Single, never been married
- A member of an unmarried couple living together or partnered

1. What is your current occupational status? Mark only one. (HINTS)

- Employed full time
- Employed part-time (multiple jobs)
- Employed pat-time (one job)
- Self-employed
- Unemployed before COVID-19
- Unemployed because of COVID-19
- Homemaker
- Student
- Retired
- Disabled
- Other (please specify)**:**________________________

1. Were you born in the United States? (HINTS)
   - Yes [If yes, go to question 25]
   - No [If no, go to question 24a]
   - Don’t Know/Not Sure [If Don’t Know/Not Sure, go to question 23a]

23a. What country were you born in?

**Write in:**___________________________________________

- Don’t Know/Not Sure

1. What is your race or ethnic background? (check all that apply)

- White
- Hispanic, Latino, or Spanish origin
- Black
- Middle Eastern or North African
- Native Hawaiian or Pacific Islander
- Asian
- American Indian, Native, First Nations, Indigenous Peoples of the Americas, or Alaska Native
- Some other Race or Origin (please specify): _________________________________________
- Don’t Know/Not Sure
- Decline to state

**Branching:** If “White” was selected, which group(s) best represents your origin or ancestry?

- - Italian
  - Irish
  - Russian
  - Polish
  - German
  - Ukrainian
  - Greek
  - French
  - Other (please specify): ________________
  - Don’t Know/Not Sure
  - Decline to State

**Branching:** If ”Hispanic, Latino, or Spanish origin” was selected, which group(s) best represents your origin or ancestry?

- - Puerto Rican
  - Dominican
  - Mexican
  - Ecuadorian
  - Colombian
  - Cuban
  - Other (please specify): ________________
  - Don’t Know/Not Sure
  - Decline to State

**Branching:** If ”Black” was selected, which group(s) best represents your origin or ancestry?

- - African American
  - Jamaican
  - Guyanese
  - Haitian
  - Trinidadian and Tobagonian
  - Nigerian
  - Ghanaian
  - Ethiopian
  - Somali
  - Other (please specify): __________________
  - Don’t Know/Not Sure
  - Decline to State

**Branching:** If “Middle Eastern or North African” was selected which group(s) best represents your origin or ancestry?

- Egyptian
- Moroccan
- Syrian
- Lebanese
- Palestinian
- Iranian
- Algerian
- Other (please specify): ____________
- Don’t Know/Not Sure
- Decline to State

**Branching:** If “Native Hawaiian or Pacific Islander” was selected, which group(s) best represents your origin or ancestry?

- Native Hawaiian
- Samoan
- Chamorro
- Tongan
- Fijian
- Marshallese
- Other (please specify): _______________
- Don’t Know/Not Sure
- Decline to State

**Branching:** If “Asian” was selected, which group(s) best represents your origin or ancestry?

- Chinese
- Asian Indian
- Filipino
- Korean
- Japanese
- Vietnamese
- Guyanese
- Bangladeshi
- Pakistani
- Other (please specify): ___________________
- Don’t Know/Not Sure
- Decline to State

**Branching:** If “American Indian, Native, First Nations, Indigenous Peoples of the Americas, or Alaska Native” was selected, which group(s) best represents your origin or ancestry?

- Iroquois or Haudenosaunee
- Blackfeet
- Cherokee
- Choctaw
- Sioux
- Central American Indian (For example, Mayan- K’iche’, Mam, Yucatan, Garifunas, among others)
- Mexican American Indian (For example, Mixteco, Nahua, Otomi, Tlapaneco, among others)
- Southern American Indian (For example, Quechua, Kichwa, Shuar, Aymara, among others)
- Other (please specify): __________________
- Don’t Know/Not Sure
- Decline to State

# Cancer beliefs

# For this next set of questions, please state your agreement with the follow statements:

1. How much do you agree or disagree with each of the following statements? (HINTS)

|  | Strongly agree | Somewhat agree | Somewhat disagree | Strongly disagree |
| --- | --- | --- | --- | --- |
| a. It seems like everything causes cancer. |  |  |  |  |
| b. There's not much you can do to lower your chances of getting cancer. |  |  |  |  |
| c. There are so many different recommendations about preventing cancer; it's hard to know which ones to follow. |  |  |  |  |

# Cancer screening knowledge

To the best of your knowledge, please answer the following questions:

1. At what age are most women supposed to start having mammograms? (HINTS)

**Write in:** ____ Years old

1. At what age are most people supposed to start doing home blood stool tests, home stool tests for colon cancer markers, having a sigmoidoscopy or having a colonoscopy? (HINTS)

**Write in:**  ____ Years old

# Cancer prevention and screening behaviors

To the best of your knowledge, please answer the following questions:

1. A blood stool test is a test that may use a special kit at home to determine whether the stool contains blood (e.g. Cologuard). Have you ever had this test using a home kit? (BRFSS)

- Yes [If yes, go to question 28a]
- No [If no, go to question 29]
- Don’t Know/Not Sure [If Don’t Know/Not Sure, go to question 30]

**28a**. How long has it been since you had your last blood stool test using a home kit? (BRFSS)

- Within the past year (anytime less than 12 months ago)
- Within the past 2 years (more than 1 year but less than 2 years ago)
- Within the past 3 years (more than 2 years but less than 3 years ago)
- Within the past 5 years (more than 3 years but less than 5 years ago)
- 5 or more years ago
- Don’t Know/Not Sure

1. Sigmoidoscopy and colonoscopy are exams in which a tube is inserted in the rectum to view the colon for signs of cancer or other health problems. Have you ever had either of these exams? (BRFSS)

- Yes [If yes, go to question 29a and 29b]
- No [If no, go to question 30]
- Don’t Know/Not Sure [If Don’t Know/Not Sure, go to question 30]

**29a.** For a SIGMOIDOSCOPY, a flexible tube is inserted into the rectum to look for problems and you are not sedated. A COLONOSCOPY is similar, but uses a longer tube, and you are usually given medication through a needle in your arm to make you sleepy and told to have someone else drive you home after the test. Was your MOST RECENT exam a sigmoidoscopy or a colonoscopy? (BRFSS)

- Sigmoidoscopy
- Colonoscopy
- Don’t Know/Not Sure

**29b.** How long has it been since you had your last sigmoidoscopy or colonoscopy? Mark only one. (BRFSS)

- Within the past year (anytime less than 12 months ago)
- Within the past 2 years (more than 1 year but less than 2 years ago)
- Within the past 3 years (more than 2 years but less than 3 years ago)
- Within the past 5 years (more than 3 years but less than 5 years ago)
- Within the past 10 years (more than 5 years but less than 10 years ago)
- 10 or more years ago
- Don’t Know/Not Sure

1. FOR FEMALES ONLY: A mammogram is an X-ray of each breast to look for breast cancer. Have you ever had a mammogram? (BRFSS)

- Yes [If yes, go to question 30a]
- No [If no, go to question 31]
- Not applicable
- Don’t Know/Not Sure [If Don’t Know/Not Sure, go to question 32]

**30a.** FOR FEMALES ONLY: How long has it been since you had your last mammogram?

- Within the past year (anytime less than 12 months ago)
- Within the past 2 years (more than 1 year but less than 2 years ago)
- Within the past 3 years (more than 2 years but less than 3 years ago)
- Within the past 5 years (more than 3 years but less than 5 years ago)
- 5 or more years ago
- Don’t Know/Not Sure

1. FOR FEMALES ONLY: A Pap test or Pap smear is a test for cancer of the cervix. Have you ever had a Pap test? (BRFSS)

- Yes [If Yes, go to question 31a]
- No [If No, go to question 32]
- Not applicable
- Don’t Know/Not Sure [If Don’t Know/Not Sure, go to question 33]

**31a**. FOR FEMALES ONLY: How long has it been since your last Pap test?

- Within the past year (anytime less than 12 months ago)
- Within the past 2 years (more than 1 year but less than 2 years ago)
- Within the past 3 years (more than 2 years but less than 3 years ago)
- Within the past 5 years (more than 3 years but less than 5 years ago)
- 5 or more years ago
- Don’t Know/Not Sure

1. FOR FEMALES ONLY: During the past three years, have you had a pelvic examination? (PhenX)

- No
- Yes, once
- Yes, more than one
- Not applicable
- Don’t know/Not Sure

1. FOR FEMALES ONLY: During the past three years, have you had an ultrasound or scan of your ovaries? (PhenX)

- No
- Yes, once
- Yes, more than one
- Not applicable
- Don’t know/Not Sure

1. FOR FEMALES ONLY: During the past three years, have you had a blood test for ovarian cancer, for example, CA-125? (PhenX)

- No
- Yes, once
- Yes, more than one
- Not applicable
- Don’t know/Not Sure

1. FOR MALES ONLY: During the past three years, have you had a digital rectal examination of the prostate? (PhenX)

- No
- Yes, once
- Yes, more than one
- Not applicable
- Don’t know/Not Sure

1. FOR MALES ONLY: A prostate-sepcific antigen test, also called a P.S.A. test, is a blood test used to check men for prostate cancer. Have you ever had a P.S.A. test? (BRFSS)

- Yes [If Yes, go to question 37a]
- No [If no, go to question 38]
- Not applicable
- Don’t know/Not Sure [If Don’t Know/Not Sure, go to question 37]

**36a**. FOR MALES ONLY: How long has it been since you had your last P.S.A. test? (BRFSS)

- Within the past year (anytime less than 12 months ago)
- Within the past 2 years (more than 1 year but less than 2 years ago)
- Within the past 3 years (more than 2 years but less than 3 years ago)
- Within the past 5 years (more than 3 years but less than 5 years ago)
- 5 or more years ago
- Don’t Know/Not Sure

1. During the past three years, have you had a chest x-ray? (PhenX)

- No
- Yes, once
- Yes, more than one
- Don’t know/Not Sure

1. Have you ever received at least one dose of the human papillomavirus (HPV) vaccine? (CHS)

- No
- Yes, one dose
- Yes, more than one dose
- Don’t Know/Not Sure

1. Have you ever received at least one dose of the hepatitis B vaccine? (NYC CHS 2018)
   - No
   - Yes, one dose
   - Yes, more than one dose
   - Don’t know/Not Sure

# Medical Mistrust

1. Please indicate how you feel about the following statements which are asking you about your feelings about the Health Care System in general:

|  | Strongly disagree | Disagree | Neither agree nor disagree | Agree | Strongly agree |
| --- | --- | --- | --- | --- | --- |
| a. The Health Care System does its best to make patients’ health better. |  |  |  |  |  |
| b. The Health Care System covers up its mistakes. |  |  |  |  |  |
| c. Patients receive high quality medical care from the Health Care System. |  |  |  |  |  |
| d. The Health Care System makes too many mistakes |  |  |  |  |  |
| e. The Health Care System puts making money above patients’ needs. |  |  |  |  |  |
| f. The Health Care System gives excellent medical care. |  |  |  |  |  |
| g. Patients get the same medical treatment from the Health Care System, no matter what the patient’s race or ethnicity. |  |  |  |  |  |
| h. The Health Care System lies to make money. |  |  |  |  |  |
| i. The Health Care System experiments on patients without them knowing. |  |  |  |  |  |

#

# Awareness of and Willingness to Participate in Clinical Trials

1. How knowledgeable would you say you are about clinical trials?

- Very knowledgeable
- Somewhat knowledgeable
- Not very knowledgeable
- Not at all knowledgeable

1. What is your opinion about clinical research among patients?

- Very positive
- Rather positive
- Neutral
- Rather negative
- Very negative

1. In your opinion, how much risk, if any is associated with participating in clinical trials?

- A lot of risk
- Some risk
- No risk at all

1. When during a cancer journey do you believe it is best to enroll in a clinical trial? (Check all that apply):
   - Immediately after diagnosis
   - In combination with the standard course of therapy
   - After all other treatment options have been exhausted
   - Never *– Selecting this option will clear your previous selections for this checkbox field*
   - Other (please specify):________________________________
2. Before today, have you ever participated in a health research study? For instance, this survey is a health research study.

- Yes
- No, this is my first
- Don’t Know/Not Sure
- Decline to State

1. Would you ever participate in another health research study?
2. Yes
3. No
4. Don’t Know/Not Sure
5. Decline to State

# Impact of COVID-19 and Reengaging Patients in Cancer Screening and Care

1. As a result of the COVID-19 pandemic, have you had to delay or cancel any routine cancer screening tests such as a mammogram, colonoscopy, lung scan, skin check, pancreatic cancer screening, or PAP/HPV test or care related to cancer?

- Yes, I chose to delay/cancel
- Yes, my provider delayed/cancelled
- No, had my tests as planned (skip to Question 52)
- N/A, was not scheduled for any (skip to Question 52)

1. How concerned are you about being behind on your cancer screening(s)?

- Very concerned
- Somewhat concerned
- Not very concerned
- Not at all concerned

1. Do you plan to reschedule your cancer screening or care?

- Yes
- No (skip to Question 52)
- Maybe

1. What is your expected timeframe for rescheduling:

- 0-3 months
- 4-6 months
- 7-9 months
- More than 9 months

1. Which type of care will you reschedule? (check all that apply):

- Office visits
- Diagnostics, testing, and lab tests
- Outpatient
- Inpatient
- Other (please specify):_________________________

1. What has motivated or what would motivate you to reschedule your care? (check all that apply)

- I am personally ready
- My provider is willing to see me
- Social distancing has been relaxed
- Provider’s COVID 19 procedures are clear
- Location is certified free of COVID-19
- My family feels comfortable
- I can afford it
- I can secure safe transportation
- Other (please specify):__________________
- None of the above *– Selecting this option will clear your previous selections for this checkbox field*

1. What are some factors that have discouraged or would discourage you from rescheduling your care? (check all that apply)

- I have no rescheduling timeline for myself
- I don’t need it
- I don’t feel comfortable
- I don’t want it
- I can’t afford it
- I have a language barrier or need help in my language
- Other (please specify):__________________
- None of the above – *Selecting this option will clear your previous selections for this checkbox field*

1. Does the vaccine mandate for health care workers in NY state make you feel more comfortable coming in for screening and routine care?

- Yes
- No
- Don’t know/Not sure

1. What are some conditions that have made or would make you feel more comfortable rescheduling? (check all that apply)

- Availability of the COVID-19 vaccine
- Sufficient personal protective equipment (PPE) for health care workers
- Regular COVID-19 testing for all staff
- Strict social distancing for patients after treatment
- No cancellation or rescheduling penalties
- Care center has given a timeline for rescheduling
- Relaxation of lockdown measures
- Option to receive service at a safer site
- Access to remote care options (e.g. virtual or telehealth)
- Safe transportation to provider’s office
- Help understanding my care in my language
- Other (please specify):__________________
- None of the above – *Selecting this option will clear your previous selections for this checkbox field*

1. Is there a type of health care site that you prefer? (check all that apply)

- Hospital
- Off-hospital clinic or office associated with a hospital
- Independent clinic or office
- Wherever my provider recommends
- Other (please specify):__________________
- None of the above – *Selecting this option will clear your previous selections for this checkbox field*

# Tobacco use

1. Have you ever smoked at least 100 cigarettes (5 packs) in your entire life? (HINTS)

- Yes
- No

1. Do you currently smoke cigarettes . . . (HINTS)

- Every day
- Some days
- Not at all

1. In the **past 7 days**, has anyone living in your home smoked one or more **cigarettes, e-cigarettes or other tobacco products**? (NYCHA Micro-Survey)

- Yes [If Yes, go to question 59a]
- No [If not, go to question 60]
- Don’t know/Not Sure [If Don’t Know/Not Sure, go to question 60]

59a. Please select which products were smoked in this home: (NYCHA Micro-Survey) (Select all that apply)

- Cigarettes
- E-Cigarettes
- Other tobacco products

# Information seeking and information access

1. Have you ever looked for information about health or medical topics from any source? (HINTS)
   - Yes
   - No
2. The most recent time you looked for information about health or medical topics, where did you go FIRST? (ONLY PICK ONE) (HINTS)

- Books
- Brochures, pamphlets, etc.
- Cancer organization
- Family
- Friend/coworker
- Doctor or health care provider
- Internet
- Library
- Magazines
- Newspapers
- Telephone information number
- Complementary, alternative, or unconventional practitioner
- Social media site, such as Facebook, Patients Like Me, Caring Bridge
- Other (please specify): __________________

1. Overall, how confident are you that you could get advice or information about health or medical topics if you needed it? (HINTS)

- Completely confident
- Very confident
- Somewhat confident
- A little confident
- Not confident at all

# Telehealth

1. Have you heard of the following terms: Telehealth, Telemedicine, or Tele-doc? (AARP)

- Yes
- No
- Don’t Know/Not Sure

# English Proficiency

1. How well do you speak English?

- Very well [If Very Well, go to question 65]
- Well [If Well, go to question 65]
- Not well
- Not at all
- Don’t Know/Not Sure

64a. Does anyone in your household over the age of ­­­14 speak English?

- Yes
- No
- Nobody in the household speaks English

1. Do you speak a language other than English at home?

- Yes
- No

65a. If yes, what language(s): ________________________

Education and Learning (OCHIN)

1. How do you learn best?

- Reading
- Listening
- Pictures
- Other (please specify): __________________
- Decline to State

Housing (OCHIN)

1. In the last month, have you slept outside, in a shelter, or in a place not meant for sleeping?

- Yes
- No
- Decline to State

1. In the last month, have you had concerns about the conditions and quality of your housing?

- Yes
- No
- Decline to State

1. In the last 12 months, how many times have you moved from one home to another? (0-30, >30)

**Write in:**____________(number of times)

Social Isolation

1. **The next questions are about how you feel about different aspects of your life. HOW MUCH OF THE TIME DO YOU FEEL… [make sure to read this prompt for each of the questions below]**

|  | Often | Some of the time | Hardly ever | Never | Don’t Know/Not Sure | Decline to State |
| --- | --- | --- | --- | --- | --- | --- |
| a. You lack companionship? | □ | □ | □ | □ | □ | □ |
| b. Left out? | □ | □ | □ | □ | □ | □ |
| c. Isolated from others? | □ | □ | □ | □ | □ | □ |
| d. That you are "in tune" with the people around you? | □ | □ | □ | □ | □ | □ |
| e. Alone? | □ | □ | □ | □ | □ | □ |
| f. That there are people you can talk to? | □ | □ | □ | □ | □ | □ |
| g. That there are people you can turn to? | □ | □ | □ | □ | □ | □ |
| h. That there are people who really understand you? | □ | □ | □ | □ | □ | □ |
| i. That there are people you feel close to? | □ | □ | □ | □ | □ | □ |
| j. Part of a group of friends? | □ | □ | □ | □ | □ | □ |
| k. That you have a lot in common with the people around you? | □ | □ | □ | □ | □ | □ |

Stress (OCHIN)

1. During the past month, how much stress would you say you experienced?

- A lot of stress
- A moderate amount of stress
- Relatively little stress
- Almost no stress at all
- Decline to State

Exposure to Violence (OCHIN)

1. Have you ever been physically or emotionally hurt or threatened by a spouse/partner or someone else you know?

- Yes
- No
- Decline to State

# Environmental Health

The field of environmental health deals with the ways in which things in our environment affect our health.

1. What are the environmental health issues in your community that concerns you the most? Please rank the top 5 issues from 1 to 5, with 1 being the highest priority and 5 being the lowest, or vice versa.

| **Concern** | **Rank** |
| --- | --- |
| Household or indoor toxins and chemicals |  |
| Food safety |  |
| Water pollution |  |
| Noise |  |
| Hazardous waste disposal |  |
| Mold |  |
| Lead |  |
| Pesticides |  |
| Drinking water quality |  |
| Outdoor air pollution (e.g, due to traffic, construction or factories/industry) |  |
| Other |  |

# Income

1. Thinking about members of your family living in your household, what is your combined annual income, meaning the total pretax income from all sources earned in the past year? (HINTS)

- $0 to $9,999
- $10,000 to $14,999
- $15,000 to $19,999
- $20,000 to $34,999
- $35,000 to $49,999
- $50,000 to $74,999
- $75,000 to $99,999
- $100,000 to $199,999
- $200,000 or more
- Don’t Know/Not Sure
- Decline to State

Cancer CHRNA Incentive Form

Thank you for participating in this survey. Please enter your information below to receive your incentive. **This information will not be linked to your survey response**. Your incentive can be mailed directly to your address, emailed to you as an electronic gift card, or sent to you via text message as a gift card code.

*If you are not interested in receiving the incentive, you do not have to fill this form out and are welcome to exit the page.*

**Please be sure to press* ***"Submit"*** *on the Cancer CHRNA survey**

1. First name: _______________________
2. Last name: _________________________
3. Date of survey completion: ______________________
4. Time of survey completion: __________________
5. How would you like to receive your incentive?
   - Email
   - Text message
   - Mailed to my home address
6. *(Branching logic- If* ***text message*** *is selected):* Cell phone: _____________________
7. *(Branching logic If* ***Mail to my home address*** *is selected):*
   1. Street address (include apartment number) _____________________
   2. City _____________________
   3. State _____________________
   4. Zip code _____________________
8. *(Branching logic if* ***Email*** *is selected):* Email address: ______________________
9. Would you be interested in being contacted to participate in other survey projects run by the NYU Langone Health Section for Health Equity?
   - Yes
   - No

Thank you for taking the survey. You should receive your incentive within the next 7-10 business days. If you have any questions, please contact [CancerCHRNA@nyulangone.org.](mailto:CancerCHRNA@nyulangone.org)

Have a nice day!
