# Supplementary material for: Prevalence of breast, cervical, and colorectal cancer screenings among select New York City populations
Source: BMC Cancer. 2025 Sep 30;25:1469. doi: 10.1186/s12885-025-14763-z (PMC12482105; doi:10.1186/s12885-025-14763-z)
Supplement: Supplementary file 2 — Supplementary Material 2. [file 12885_2025_14763_MOESM2_ESM.docx]

Additional file 2

| **Race and Ethnicity** |  |
| --- | --- |
| **Questions** | **Response options (check all that apply)** |
| *Cancer CHRNA* |  |
| What is your race or ethnic background? (check all that apply) | White; Hispanic, Latino, or Spanish origin; Black; Middle Eastern or North African; Native Hawaiian or Pacific Islander; Asian; American Indian, Native American, First Nations, Indigenous People of the Americas, or Alaska Native; Some other Race or Origin (please specify); Don’t know/Not sure; Decline to state |
| If "White" was selected, which group(s) best represents your origin or ancestry? | Italian; Irish; **Russian; Polish;** German; **Ukrainian;** Greek; French; Other (please specify); Don’t know/Not sure; Decline to state |
| Other Eastern European ethnicities included for categorization | Belarusian; Dagestan; Moldovan |
| If "Hispanic, Latino, or Spanish origin" was selected, which group(s) best represents your origin or ancestry? | Puerto Rican; Dominican; Mexican; Ecuadorian; Colombian; Cuban; Other (please specify); Don’t know/Not sure; Decline to state |
| If 'Black' was selected, which group(s) best represents your origin or ancestry? | African American; **Jamaican; Guyanese; Haitian; Trinidadian and Tobagonian**; Nigerian; Ghanaian; Ethiopian; Somali; Other (please specify); Don’t know/Not sure; Decline to state |
| Other Caribbean ethnicities included for categorization | Caribbean; Dominican; St. Lucian; and St. Vincent & Grenada |
| If "Middle Eastern or North African" was selected, which group(s) best represents your origin or ancestry? | Egyptian; Moroccan; Syrian; Lebanese; Palestinian; Iranian; Algerian; Other (please specify); Don’t know/Not sure; Decline to state |
| Other SWANA ethnicities included for categorization | Afghani; Iraqi; Israeli; Jordanian; Saudi; Yemeni |
| If "Asian" was selected, which group(s) best represents your origin or ancestry? | Chinese; Asian Indian; Filipino; Korean; Japanese; Vietnamese; Guyanese; Bangladeshi; Pakistani; Other (please specify); Don’t know/Not sure; Decline to state |
| South Asian ethnicities and countries of birth | Bangladeshi, Pakistani, Asian Indian, and Nepali |
| Western/Central Asian ethnicities for categorization | Armenian; Azerbaijani; Georgian; Kazakh; Latvian; Lithuanian; Tajik; Uzbek |
| *CHS (2017-2020)* |  |
| Are you Hispanic or Latino? Which one or more of the following would you use to describe yourself as… | White, non-Hispanic; Black, non-Hispanic; Hispanic; Hispanic; Asian/Pacific Islander, non-Hispanic; North African/Middle Eastern, non-Hispanic (2019 & 2020); Other, non-Hispanic |
| Please tell me which group represents your Hispanic or Latino origin or ancestry. | Puerto Rican; Dominican/Dominican American; Mexican/Mexican American; Ecuadorian; Colombian; Cuban/Cuban American; Central American/ Other Central American; South American/ Other South American; (VOL) Spanish; Other Latin American/ Hispanic/ Latino or Something else; Don’t know; Refused; Missing |
| Please tell me which group represents your Asian heritage or ancestry. | Chinese; Asian Indian; Filipino; Korean; Japanese; Vietnamese; Something else (specify); Don’t know; Refused; Missing |
| Please tell me which group represents your Asian heritage or ancestry. | East Asian; South Asian; Southeast Asian; Other; Don’t know; Refused; Missing |
| Some people in addition to being Black, have a certain heritage or ancestry. Do you identify with any of these? (2019 & 2020) | African American; Caribbean or West Indian; A recent immigrant or the child of recent immigrants from Africa; Other Black heritage or ancestry; Does not identify with any of these; Don’t know; Refused; Missing |
| SWANA countries of birth | Algeria, Egypt, Iraq, Iran, Israel, Jordan, Kuwait, Lebanon, Libya, Morocco, Oman, Palestine, Saudi Arabia, Syria, Tunisia, United Arab Emirates, Yemen, and ‘Middle Eastern’ |
| Central or Western Asia countries of birth | Central Asia, Uzbekistan, Tajikistan, Kazakystan, Krygzstan, Armenia, Azerbaijan, Georgia, Lithuania, Latvia, or Estonia |
| Eastern European countries or birth | The former USSR, Russia, Ukraine, Poland, Moldova, Belarus, Hungary, Slovakia, Czechoslovakia, Romania |
| Caribbean countries of birth | Haiti, Trinidad and Tobago, Antigua, Barbados, Dominica, Grenada, Jamaica, St. Martin, Caribbean, Bahamas, Virgin Islands, St. Vincent, St. Lucia, West Indian, Guyana, Cayman Islands, Virgin Islands, St. Croix, and St. Kitts and Nevis |
| **Colon Cancer** |  |
| *Cancer CHRNA* |  |
| A blood stool test is a test that may use a special home kit to determine whether the stool contains blood (e.g., Cologuard). Have you ever had this test using a home kit? | Yes; No; Don’t know/Not sure |
| How long has it been since you had your last blood stool test using a home kit? | Within the past year (anytime less than 12 months ago); Within the past 2 years (more than 1 year but less than 2 years ago); Within the past 3 years (more than 2 years, but less than 3 years ago); Within the past 5 years (more than 3 years, but less than 5 years ago); 5 or more years ago; Don't Know/Not Sure |
| Sigmoidoscopy and colonoscopy are exams in which a tube is inserted in the rectum to view the colon for signs of cancer or other health problems. Have you ever had either of these exams? | Yes; No; Don’t know/Not sure |
| For a SIGMOIDOSCOPY, a flexible tube is inserted into the rectum to look for problems. A COLONOSCOPY is similar, but uses a longer tube, and you are usually given medication through a needle in your arm to make you sleepy and told to have someone else drive you home after the test. Was your MOST RECENT exam a sigmoidoscopy or a colonoscopy? | Sigmoidoscopy; Colonoscopy; Don’t know/Not sure |
| How long has it been since you had your last sigmoidoscopy or colonoscopy? Mark only one. | Within the past year (anytime less than 12 months ago); Within the past 2 years (more than 1 year but less than 2 years ago); Within the past 3 years (more than 2 years, but less than 3 years ago); Within the past 5 years (more than 3 years, but less than 5 years ago); Within the past 10 years (more than 5 years, but less than 10 years ago); 10 or more years ago; Don't Know/Not Sure |
| *CHS (2018-2020)* |  |
| Colonoscopy is an exam in which a tube is inserted in the rectum to view the bowel for signs of cancer or other health problems. Have you ever had a colonoscopy? | Yes; No; Don’t know; Refused |
| When was your most recent colonoscopy performed? | Less than 1 year ago; 1 year but less than 5 years ago; 5 years ago but less than 10 years ago; 10 or more years ago; Don’t know/Not sure; Refused |
| **Breast Cancer (FOR FEMALES ONLY)** |  |
| *Cancer CHRNA* |  |
| A mammogram is an X-ray of each breast to look for breast cancer. Have you ever had a mammogram? | Yes; No; Not applicable; Don’t know/Not sure |
| How long has it been since you had your last mammogram? | Within the past year (anytime less than 12 months ago); Within the past 2 years (more than 1 year but less than 2 years ago); Within the past 3 years (more than 2 years, but less than 3 years ago); Within the past 5 years (more than 3 years, but less than 5 years ago); 5 or more years ago; Don't Know/Not Sure |
| *CHS (2019 only)* |  |
| A mammogram is an x-ray of each breast to look for breast cancer. Have you ever had a mammogram? | Yes; No; Don’t know; Refused |
| How long has it been since your last mammogram? | Less than 12 months ago; 1 year but less than 2 years ago; 2 years but less than 3 years ago; 3 years but less than 5 years ago; 5 or more years ago; Don’t know/Not sure; Refused |
| **Cervical Cancer (FOR FEMALES ONLY)** |  |
| *Cancer CHRNA* |  |
| A Pap test or Pap smear is a test for cancer of the cervix. Have you ever had a Pap test? | Yes; No; Not applicable; Don’t know/Not sure |
| How long has it been since your last Pap test? | Within the past year (anytime less than 12 months ago); Within the past 2 years (more than 1 year but less than 2 years ago); Within the past 3 years (more than 2 years, but less than 3 years ago); Within the past 5 years (more than 3 years, but less than 5 years ago); 5 or more years ago; Don't Know/Not Sure |
| *CHS (2017 only)* |  |
| A Pap smear is a test for cancer of the cervix. Have you ever had a Pap smear? | Yes; No; Don’t know; Refused |
| How long has it been since your last pap smear? | Less than 12 months ago; 1 year but less than 2 years ago; 2 years but less than 3 years ago; 3 years but less than 5 years ago; 5 or more years ago; Don’t know/Not sure; Refused |
