# Supplementary material for: Prevalence of breast, cervical, and colorectal cancer screenings among select New York City populations
Source: BMC Cancer. 2025 Sep 30;25:1469. doi: 10.1186/s12885-025-14763-z (PMC12482105; doi:10.1186/s12885-025-14763-z)
Supplement: Supplementary file 3 — Supplementary Material 3. [file 12885_2025_14763_MOESM3_ESM.docx]

Additional file 3. Adjusted relative risk of up-to-date CRC screening among individuals ages 50-75, RR (95% CI)

|  | Cancer CHRNA 2021-2022 | |
| --- | --- | --- |
|  | Model 1 | Model 2 |
| Race and Ethnicity |  |  |
| Eastern European | 1 [Reference] | 1 [Reference] |
| Afro-Caribbean | **0.57 (0.41-0.79)** | **0.62 (0.45-0.86)** |
| Latina/o | **0.62 (0.49-0.78)** | **0.77 (0.60-0.98)** |
| Chinese | **0.81 (0.70-0.94)** | 0.85 (0.72-1.01) |
| Korean | **0.61 (0.48-0.78)** | **0.72 (0.57-0.90)** |
| South Asian | **0.50 (0.36-0.71)** | **0.54 (0.38-0.76)** |
| SWANA | **0.64 (0.51-0.80)** | **0.68 (0.53-0.86)** |

Abbreviations: SWANA, Southwest Asian and North African; CHRNA, Cancer Community Health Resources and Needs Assessment; RR, relative risk; CI, confidence interval

Bolded values are significant at p<0.05

CHS data includes individuals aged 50-74

Model 1: adjusted for age group and sex

Model 2: adjusted for age group, sex, income, education, health insurance, check-up <1 year ago, nativity, and English spoken fluency
